# Supplementary material for: Size Matters? A Comprehensive In Vitro Study of the Impact of Particle Size on the Toxicity of ZnO
Source: Nanomaterials (Basel). 2023 Jun 4;13(11):1800. doi: 10.3390/nano13111800 (PMC10255545; doi:10.3390/nano13111800)
Supplement: Supplementary file 1 [file nanomaterials-13-01800-s001.zip › nanomaterials-2426191-supplementary.pdf]

Supplementary Materials

Size Matters? A Comprehensive *in vitro* study/investigation of the impact of particle size on the toxicity of ZnO

Montserrat Mitjans [1,2], Laura Marics [1], Marc Bilbao [1], Adriana S. Maddaleno [1], Juan José Piñero [1] and M. Pilar Vinardell [1,2,*]

[1] Physiology, Dpt. Biochemistry and Physiology, Universitat de Barcelona. montsemitjans@ub.edu; laura.marfa.89@gmail.com; m.bilbasen@gmail.com; adrianamaddaleno@ub.edu; juanjopinero@ub.edu;

[2] Institute of Nanoscience and Nanotechnology, Universitat de Barcelona

* Correspondence: mpvinardellmh@ub.edu

1.- Energy-dispersive X-Ray spectroscopy (or analysis) (EDX) and selected area diffraction analysis (SAED)

Besides to observation of NPs, an elementary analysis by Energy-dispersive X-Ray spectroscopy (or analysis) (EDX) was performed in all conditions studied. This technique can offer information about the chemical nature of the aggregates observed. From the spectra obtained, we identified the characteristic band of Zn at 8.5 keV and another one at 0.5 keV, which identifies oxygen. These two bands are observed in all spectra independently of the media used therefore it is suggested that the aggregates can be attributed to ZnO. As an example, Figure S1 shows the spectrum obtained after the elemental analysis of an aggregate of ZnO 100 nm in ethanol.

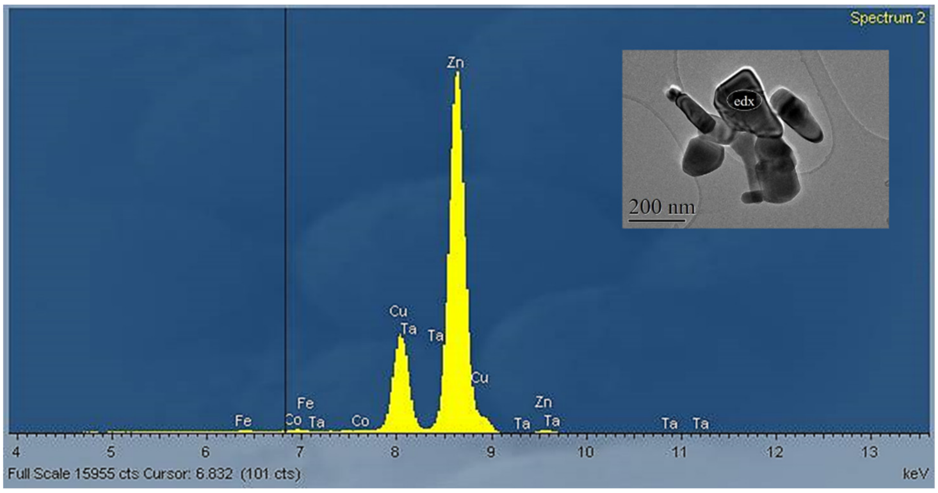

Figure S1. Example of EDX elemental analysis for ZnO 100 nm in ethanol used to confirm the chemical nature of the aggregates observed.

Also, we have measured the distance between lattice planes from a Selected Area Diffraction (SAED) of the different aggregates of ZnO in different media is analyzed, by using a diffraction pattern or high-resolution photograph, as shown in Figure S2 as an example. There are two ways to calculate the lattice distances, using a diffraction pattern (Figure S2B) or high-resolution photograph (Figure S2C). The experimental values obtained (Table S1) confirmed that the compound is ZnO and that their structure is hexagonal by both methods.

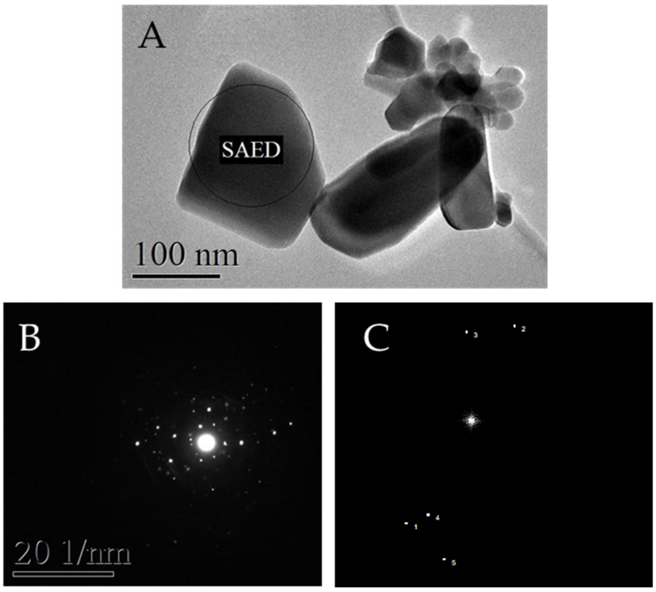

Figure S2. Example of selected area diffraction analysis (SAED) for ZnO 100 nm in ethanol used to confirm the chemical nature of the aggregates observed. A: aggregate and area selected; B: diffraction pattern; C: high-resolution photograph that allows to measure the lattice distance between two points.

Once such dimensions are obtained, they are compared with the power diffraction pattern database maintained by ICDD (International Centre Diffraction Data, previously known as JPCDS or Joint Committee on Powder Diffraction Standards. This database is used by the "Centres Científics I Tecnològics de la Universitat de Barcelona", within the program PCPDFWIN.

Table S1. Values of theoretical and experimental lattice distance and their Miller indexes.

Point	Calculated d-spacing (Å)	Standard d-spacing (Å)	Miller index
1	2.090	1.9112	[102]
2	2.449	2.4760	[101]
3	2.874	2.8146	[100]
4	2.444	2.4760	[101]
5	1.796	1.6250	[110]

2.- Dynamic Light Scattering (DLS)

The average hydrodynamic diameter of the nanoparticles (ZnO 50 and 100 nm) was determined using a Malvern Zetasizer Nano ZS. The size analyses were performed at a scattering angle of 173°, after dilution in PBS at 100 µg/mL and placed in ultrasonic bath for 10 min. Results are presented in Table S.2 and Figure S.2.

Table S2. DLS characterization of nanoparticles at room temperature.

ZnO particles	HD (nm)[1]	PdI
50 nm	1,699 ± 239.3	0.426
100 nm	2,121 ± 45.88	0.851

[1]Results are expressed as mean ± standard error (n=3)

3.-Hemoglobin spectra after incubation with ZnO.

Figure S3 on the left shows human hemoglobin (a), denaturalized hemoglobin (b) and oxidized hemoglobin (c) spectra. Denaturalization induces a decrease of the typical peaks of human hemoglobin (black arrows), whereas the presence of an oxidant agent as $K_3Fe(CN)_6$ not only alters these peaks also a novel peak at approximately 630 nm is recorded. The effect of ZnO is presented on the right. The exposure of erythrocytes to different concentrations of ZnO causes their lysis and, consequently, the release of hemoglobin into the medium. This release is proportional to the hemolysis produced but the spectrum still shows the typical hemoglobin peaks (1-5). However, at the maximal concentration assayed (6), ZnO interacts with hemoglobin protein causing the disappearance of the 540 and 575 nm peaks and an increase of absorbance at 630 nm indicating that hemoglobin is being oxidized.

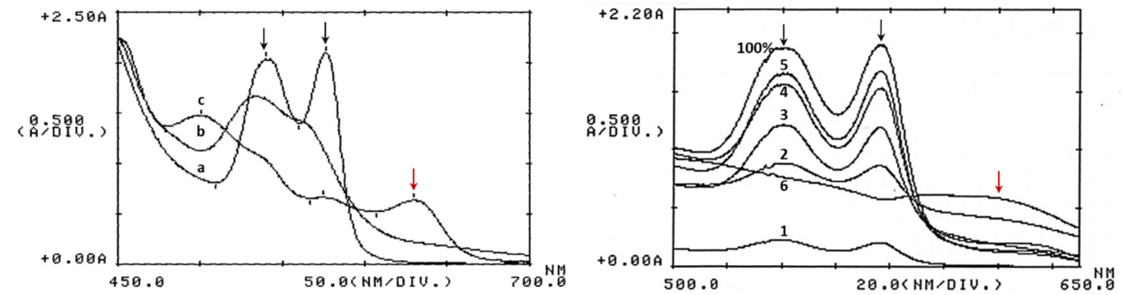

Figure S3. Spectra of human hemoglobin. Left: spectra Human hemoglobin (a) and denaturalized and oxidized human hemoglobin (b and c) spectra. Hemoglobin denaturalization was induced by addition of 1% of sodium dodecyl sulfate in PBS pH 7.4 while the presence of $K_3Fe(CN)_6$ causes total hemoglobin oxidation. Right: hemoglobin released after 24 hours incubation of RBC in the presence of different concentrations (1-6) of ZnO at 37°C. 1-6: 0.2, 0.4, 0.8, 1 and 2 mg/mL. 100%: typical spectrum of human after hemoglobin. Black arrows indicate 540 nm and 575 nm peaks whereas red arrow indicates the 630 nm peak.

4.- Formation of protein corona

The huge protein concentration in plasma, estimated to be about 7.6 mg/mL, is basically represented by a few abundant proteins as albumin or fibrinogen making difficult to detect low abundance proteins that represent 1% of the total protein amount [1, 2]. However, as presented in Table S3, an approximate molecular weight could be assigned to the stained bands when compared with the molecular weight standard. Because the intensity of stained bands is directly related to protein abundance, we have identified potential protein candidates in the lane corresponding to human plasma (Lane 1, Figure 11.B).

Table S3. Qualitative staining intensity of SDS-PAGE protein bands and potential plasma protein candidates.

SDS-PAGE MW band[1]	Qualitative staining intensity[2]				Potential candidates[3]		
	Human plasma	50 nm	100 nm	micro	Protein	g/L	kDa
46	+++	++	++	+	Fibrinogen γ	2.0-4.0 × 10^{-3}	46-49 [2, 4, 5]
52	+++	++	++	+	Factor VII<br>Fibrinogen β<br>α1-antitripsin	1.0 × 10^{-6}<br>2.0-4.0 × 10^{-3}<br>7.8-20 × 10^{-4}	50-53 [1, 2]<br>52-56 [2, 4, 5]<br>54 [1]
66	+++	++	++	++	Fibrinogen α<br>Albumin<br>Antithrombin II	2.0-4.0 × 10^{-3}<br>3.5-5.2 x 10^{-2}<br>2.0 × 10^{-4}	66 [4, 5]<br>66-69 [1, 2, 3]<br>65 [3]
≈ 70	++	++	+	+	Prothrombin	1.0 × 10^{-4}	70 [2]
≈ 75	++	-	+	+	Transferrin	0.8-3.6 × 10^{-6}	77-80 [2, 3]
90	+	+	-	-	Plasminogen	1.5-3.5 × 10^{-4}	81-90 [2, 3]
110	+	-	-	-	Haptoglobin	3.0-22 × 10^{-4}	100 [1]
135	+	+	+	-	Ceruloplasmin	1.5-6.0 × 10^{-4}	135 [3]
150	+	+	-	-	Immunoglobulin G	0.7-1.6 x 10^{-2}	150 [1]
160	++	-	-	-	Immunoglobulin A	7.0-40 × 10^{-4}	160 [1]

[1]Aproximate molecular weight band identified according to molecular weight marker. [2]Staining intensity: High: +++; Significant: ++; Slight: +; Apparently absent: -; [3]According to protein abundance and molecular weights described elsewhere [1-5].

Finally, protein presence was corroborated by elemental analysis (EDX) from the same TEM instrument used to obtain the images of NPs. As an example, Figure S4 shows the obtained EDX from Figure 12B where a shadowy staining around ZnO 50 nm particles reported presence of tungsten.

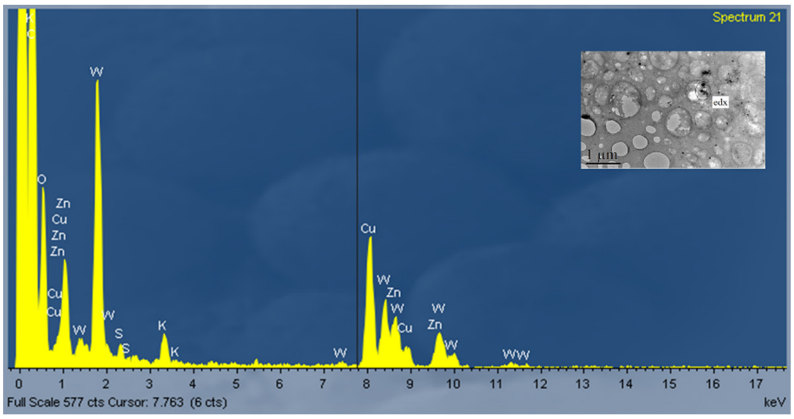

Figure S4. Example of EDX elemental analysis for ZnO 50 nm after 30 minutes incubation in human plasma to confirm the presence of protein corona. Particles were suspended in MiliQ water and one sample drop was deposited and dried on a Holey Carbon- Cu grid and protein were revealed by adding phosphotungstic acid. EDX (Energy-dispersive X-ray spectroscopy) analyses were performed to analyze the elemental composition of the sample, thus confirming the presence of plasma proteins, ZnO particles and the staining agent (tungsten, from the phos-photungstic acid). Observation and analysis were carried out in a JEOL JEM LaB6-2100 micro-scope at CCiT UB.

References

1- Lundblad, R.; Considerations for the Use of Blood Plasma and Serum for Proteomic Analysis. *The Internet Journal of Genomics and Proteomics*. **2003**, 1(2):1-8.

2- Schenk, S.; Schoenhals, G.J.; de Souza, G.; Mann, M.; A high confidence, manually validated human blood plasma protein reference set. *BMC Medical Genomics* **2008**, 1:41 doi:10.1186/1755-8794-1-41.

3- Ahmed F.E.; Sample preparation and fractionation for proteome analysis and cancer biomarker discovery by mass spectrometry. *J. Sep. Sci.* **2009**, 32:771-798. doi 10.1002/jssc.200800622.

4- Mahmoudi, M.; Abdelmonem, A.M.; Behzadi,S.; Clement, J.H.; Dutz, S.; Ejtehadi, M.R.; Hartmann, R.; Kantner, K.; Linne, U.; Maffre, P.; Metzler, S.; Moghadam M.K.; Pfeiffer, C.; Rezaei, M.; Ruiz-Lozano, P.; Serpooshan, V.; Shokrgozar, M. A.; G. Ulrich Nienhaus, G.U.; Parak W.J.; Temperature: The "Ignored" Factor at the NanoBio Interface. *ACS Nano*. **2013**, 7(8):6555-62. doi: 10.1021/nn305337c

5- Tennent, G.A.; O. Brennan, S.O.; Stangou, A.J.; O'Grady, J.; Hawkins, P.N.; Pepys M.B; Human plasma fibrinogen is synthesized in the liver. *Blood*. **2007**, 109 (5):1971–1974. https://doi.org/10.1182/blood-2006-08-040956.

6- Tan, Y.J.; Tham, P.Y.; Chan, D.Z.L.; Chou, C.F.; Shen, S.; Burtram C. Fielding, B. C.; Tan, T.H.P.; Lim, S. G.; Hong, W.; The Severe Acute Respiratory Syndrome Coronavirus 3a Protein Up-Regulates Expression of Fibrinogen in Lung Epithelial Cells. *J. Virol.* **2005**, 79(15): 10083–10087. doi:10.1128/JVI.79.15.10083–10087.2005
